# Clinical Characteristics of Newborns Born to Mothers with COVID-19

**DOI:** 10.3390/jcm10194383

**Published:** 2021-09-25

**Authors:** Katarzyna Wróblewska-Seniuk, Agnieszka Basiukajć, Dobrochna Wojciechowska, Mayanthi Telge, Izabela Miechowicz, Jan Mazela

**Affiliations:** 1 Department of Newborns’ Infectious Diseases, Poznan University of Medical Sciences, 60-535 Poznan, Poland; aga_basiukajc90@wp.pl (A.B.); dobrochna.naskrecka@gmail.com (D.W.); janco@pol-med.com.pl (J.M.); 2 Student Research Club at the Department of Newborns’ Infectious Diseases, Poznan University of Medical Sciences, 60-535 Poznan, Poland; mayanthi.dilshani@gmail.com; 3 Department of Informatics and Statistics, Poznan University of Medical Sciences, 60-535 Poznan, Poland; iza@ump.edu.pl

**Keywords:** newborns, SARS-CoV-2, COVID-19, infection, pregnancy, respiratory insufficiency, respiratory distress, transient tachypnea of the newborn

## Abstract

(1) Background: According to the literature, most outcomes of neonates born to mothers infected with SARS-CoV-2 are favorable. This study aimed to assess the clinical characteristics of newborns born to infected women in a tertiary center in Poznan, Poland. (2) Methods: The study comprised 101 newborns delivered by women infected with SARS-CoV-2. The control group consisted of 101 newborns born before the pandemic. Data were collected retrospectively from the medical records. (3) Results: Most newborns of SARS-CoV-2-positive mothers were delivered by cesarean section—83.17% vs. 40.59% in the control group (*p* < 0.05). The groups did not differ in Apgar scores and the need for resuscitation. Newborns of SARS-CoV-2-positive mothers were more likely to present with respiratory distress and require respiratory support. The most common diagnosis was transient tachypnea of the newborn, not correlated with the mode of delivery. Newborns of the study group were never exclusively breastfed, 0% vs. 64.36% (*p* < 0.05). None of the patients in the study group was tested positive for the virus. (4) Conclusions: Infants born to SARS-CoV-2-positive mothers seem to be more at risk of moderate respiratory failure than other newborns. Separation of mother–baby dyads results in a dramatic fall in breastfeeding in the short-term post-partum period.

## 1. Introduction

In December 2019, a newly identified coronavirus (Severe Acute Respiratory Syndrome Coronavirus 2, SARS-CoV-2) caused an outbreak of coronavirus disease (COVID-19) in Wuhan, in Hubei Province, China, and has since then been spreading worldwide [1]. On 30 January 2020, the World Health Organization (WHO) declared the outbreak of COVID-19 as a Public Health Emergency of International Concern [2]. So far as we know, people of all ages are susceptible to SARS-CoV-2, while the elderly and those with underlying diseases are more fragile to the virus. To date, hundreds of pediatric cases have been documented, including neonatal infection. This situation has drawn the public’s attention to neonatal infection [3].

SARS-CoV-2 may spread from person to person by droplets from respiratory secretions. The infection may also occur through contact with an infected surface when the person touches the eyes, nose, or mouth. Airborne protections are generally recommended when aerosol-generating procedures are performed [4]. The incubation period of SARS-CoV-2 infection ranges from 1 to 14 days, with an average of 3 to 7 days.

Many retrospective studies have been conducted to analyze the implications of COVID-19 in pregnant women on their newborns. It is known that vertical transmission of SARS-CoV-2 is possible but seems to occur in a minority of cases of maternal coronavirus disease in the third trimester [5,6]. Many precautionary measures have been recommended, including isolating the newborn from the mother, refraining from breastfeeding, and early washing of the newborns [7]. Generally, all outcomes of neonates born to mothers positive for COVID-19 are favorable, with any severe symptoms being attributed to prematurity due to elective cesarean section of all mothers with a positive Real-Time Polymerase Chain Reaction (RT-PCR) assay [3,8,9]. 

This study aimed to assess the clinical characteristics and potential risk of pregnant women with COVID-19 on their babies to provide an experience for the prevention and treatment of newborns born to mothers infected with SARS-CoV-2.

## 2. Materials and Methods

From May to December 2020, 101 newborns were delivered by pregnant women infected with SARS-CoV-2 in late pregnancy in the Gynecological-Obstetrical Hospital of Poznan University of Medical Sciences, Poznan, Poland. Some patients had signs and symptoms of a viral infection such as fever, dyspnea, and cough, and others had contact with someone infected with SARS-CoV-2. Some were diagnosed with SARS-CoV-2 on admission to the hospital, where the antigen test was performed for screening. In all patients, the diagnosis of SARS-CoV-2 infection was confirmed before delivery by the pharyngeal swab and the RT-PCR test. Women diagnosed with SARS-CoV-2 were admitted to the department designated for patients with clinical suspicion or confirmed infections with SARS-CoV-2.

After delivery, all newborns were immediately transferred to the Department of Newborns’ Infectious Diseases for isolation and observation. They were isolated in neonatal incubators and monitored continuously. A detailed examination was performed with respiratory precautions. Vital signs, including body temperature, respiratory rate, heart rate, and oxygen saturation, were measured. Antibiotics were not used unless signs of bacterial infection during hospitalization occurred. Pharyngeal swabs were collected on the second day of life. They were sent to the hospital laboratory for nucleic acid extraction and tested for SARS-CoV-2 by real-time polymerase chain reaction (RT-PCR detection kit Gene Xpert Xpress SARS-CoV-2 (CEPHEID, Sunnyvale, CA, USA).

Newborns were discharged home as soon as possible when their condition was stable and continued with home isolation. Parents were informed about possible signs and symptoms of COVID-19 infection and instructed where to call in case of emergency. We followed up on all the newborns by a telephone call within 3–4 weeks after discharge from the hospital to check if any problems occurred and learn about their feeding, sanitary regime at home, and potential complications and hospitalizations.

The mothers’ demographic data, pregnancy and delivery history, neonatal birth status including Apgar score and clinical features, and SARS-CoV-2 nucleic acid detection results in the neonatal pharyngeal swabs were collected. The clinical characteristics of newborns and perinatal complications were analyzed.

The control group of 101 newborns was selected from newborns born in the same hospital and from the comparable period of the year 2019, before the outbreak of the SARS-CoV-2 pandemic. Data for this group were collected retrospectively, based on the medical charts and hospital electronic documentation. The group was selected considering the newborn’s maturity as well as pregnancy complications by means of the propensity score-matching method. The logistic regression model was built to use this method, considering such variables as a week of gestation at delivery, premature rupture of membranes, group-B streptococcus colonization in mothers, hypertension, and diabetes during pregnancy.

The normality of the distribution of variables was assessed using Shapiro-Wilk’s test. Student’s t-test was used for variables with normal distribution and equal variance to compare variables between groups. The Mann–Whitney test was used for variables without normal distribution and those measured on an ordinal scale. The chi-squared test of independence or Fisher’s exact test was used to measure the relationship between categorical variables. The odds ratio (OR) was calculated with a 95% confidence interval (CI) if there was a relation.

Newborns of the control group were hospitalized in rooming-in wards and were admitted to the neonatal pathology unit only if they required surveillance or treatment for particular conditions. We followed up on this group’s patients by telephone call to collect data about their health status and feeding method in the neonatal period.

The Medical Ethical Committee of Poznan University of Medical Sciences approved this study.

Statistical analysis was performed with Statistica 13 (TIBCO, Palo Alto, CA, USA). The results were interpreted as statistically significant when *p* < 0.05.

## 3. Results

The study and control group consisted of late-preterm and term infants (Table 1). The groups did not differ in pregnancy complications such as hypertension, diabetes, or intrauterine infection; however, intrauterine growth restriction (IUGR) was observed significantly more frequently in the control group than in the COVID-19 group (OR = 10.989, 95% CI 1.379–87.58) (Table 1). Most newborns of SARS-CoV-2-positive mothers were delivered by cesarean section (83.17% vs. 40.59%, *p* < 0.05).

The groups did not differ in Apgar scores and the need for resuscitation in the delivery room (Table 2). Newborns of SARS-CoV-2-positive mothers were three times more likely to present with respiratory distress in the first few days of life (OR= 3.016, 95% CI: 1.128 to 8.0633). It was identified in 16 (16.0%) newborns of SARS-CoV-2-positive mothers and only in 6 (5.94%) infants of the control group (*p* = 0.02). The most common diagnosis was transient tachypnea of the newborn, and the risk of this pathology in newborns of SARS-CoV-2-positive mothers was three times higher than in the control group (OR= 3.270, 95% CI: 1.017 to 10.512) (Table 3). 

Using the Fisher-Freeman-Halton test, we checked whether there was an association between the mode of delivery and the respiratory distress or TTN alone in the analyzed groups. We did not find any correlation (Table 4).

Respiratory support was necessary for 16 (16.0%) newborns of SARS-CoV-2-positive mothers and only 4 (3.96%) infants of the control group (*p* = 0.01). Seven patients (6.93%) required non-invasive ventilation (NIV), 8 (7.92%) were treated with nasal CPAP (nCPAP), and 1 (0.99%) required passive oxygen supplementation. In the control group, 4 newborns (3.96%) needed nCPAP. The mean time of respiratory support in the study group was 9.34 h (max. 264 h), and in the control group, it was only 0.58 h (max. 32 h) (*p* < 0.01).

Other symptoms observed in infants of SARS-CoV-2-positive mothers were feeding intolerance in 2 (1.98%) patients vs. 1 (0.99%) in the control group (NS) and intraventricular hemorrhage of grade I in 1 patient (0.99%).

We also analyzed the method of feeding infants during the hospital stay. None of the newborns of SARS-CoV-2-positive mothers were exclusively breastfed 0% vs. 64.36% in the control group (*p* < 0.01). Thirty-one newborns (30.69%) of the study group were fed with formula and expressed mother’s milk (vs. 34; 33.66% in the control group), and 70 (69.31%) were fed exclusively with formula (vs. 2; 1.98% in the control group, *p* < 0.01).

None of the symptomatic patients in the study group was tested positive for the virus.

The duration of hospitalization was similar in the study group and the control group. In the study group, the hospital stay lasted approximately 3.39 days (max. 22 days), and in the control group, it was 3.59 days (max. 17 days).

## 4. Discussion

Many studies have been conducted to analyze the relationship between COVID-19 in pregnant women and its implications on their newborns. It is known that vertical transmission of SARS-CoV-2 is possible but seems to occur in a minority of cases of maternal coronavirus disease in the third trimester. Many papers have reported an aggravated course of pregnancy [8]. However, neonates present mostly with a favorable prognosis or with signs and symptoms of respiratory distress [10,11].

The clinical characteristics of COVID-19 in infected mothers and their neonates were first assessed by Chen et al. in a retrospective study [12]. All neonates in this study were born by cesarean section, and all presented with negative results for COVID-19 in all tested samples, including neonatal throat swabs, cord blood, and amniotic fluid. The authors reported fetal distress in two of the nine newborns. 

A study conducted by Zhu et al. also evaluated the clinical features of neonates born to mothers positive for COVID-19 [13]. Newborns presented with fetal distress, respiratory distress, thrombocytopenia, abnormal liver function tests, and even the death of one patient occurred. The most common symptoms were dyspnea, fever, tachycardia, and vomiting. Seven out of nine neonates had abnormal chest computer tomography (CT) with the picture of neonatal respiratory distress syndrome (2), infection (4), and pneumothorax (1). Intrauterine fetal distress was observed in 6 out of 9 cases in this study. Zhu and colleagues speculate this is due to the hypoxemia caused by the infection that leads to birth asphyxia and premature birth [13]. Pregnant women generally have a reduced tolerance to hypoxia, which is exacerbated by any superimposed viral infection. This poses significant threats to both mother and child, causing fetal distress leading to emergency cesarean sections [14].

In our analysis, none of the infants was found positive for SARS-CoV-2. We did not observe any severe course of the disease in newborns, either. Most infants were asymptomatic and were discharged home as healthy newborns. In 16% of patients, we observed symptoms of respiratory distress and the need for respiratory support.

Apgar scores in our study group patients did not differ significantly from the control group, which is in accordance with observations of other researchers [9,15,16,17]. Resuscitation in the delivery room was required in 4 newborns, which did not differ from the control group, either.

Most researchers suggest that any symptoms observed in newborns born to SARS-CoV-2-positive women result from the elective cesarean section, which is frequently performed just because of COVID-19 infection. In our study group, most infants were born by cesarean section (83.17%). Its rate was significantly higher than in the control group. However, we did not find any association between delivery mode and the need for resuscitation or respiratory distress. Thus, we can conclude that higher respiratory distress rates and the need for respiratory support in newborns resulted only from COVID-19 infection in the mother. However, its mechanism is not easily explicable.

The most frequent form of respiratory insufficiency in our study group was transient tachypnea of newborns. Another was respiratory distress syndrome, requiring surfactant administration and pneumonia. All these patients required respiratory support in the form of nCPAP or non-invasive ventilation, the duration of which was longer when compared to the babies born to non-infected mothers. These results are consistent with the data reported by Yang et al. They reinforced the necessity of nCPAP to relieve the symptoms of mild respiratory distress syndrome and mild grunting [18]. Similarly, Hu et al. reported the necessity of nCPAP for 7 and 23 days in two newborns due to prematurity, resulting in respiratory distress syndrome and apnea of prematurity [10]. Vardhelli et al. postulated that newborns’ respiratory symptoms are most likely due to prematurity, transient tachypnea, and respiratory distress rather than due to COVID-19 pneumonia [6]. These results are in line with our research.

Dumitriu et al. evaluated the outcomes in neonates born to COVID-19-positive mothers in the first 6 weeks of the pandemic [7]. These newborns’ clinical characteristics were similar to newborns born to non-infected mothers, except for the higher rate of newborn hyperbilirubinemia and higher risk of premature birth [7]. We did not confirm these observations in our study.

In our study, it was noteworthy that intrauterine growth restriction (IUGR) was more frequent in newborns of the control group born to non-infected mothers. This finding is rather peculiar and difficult to explain. IUGR is attributed mainly to pregnancy complications, such as smoking, severe diabetes or hypertension in mothers, placental flow disturbances, and inflammation [10]. However, the incidence of all these factors was similar in both groups since the control group was collected considering pregnancy complications. On the other hand, we should not expect the influence of viral infection on fetal growth since our study group consisted only of newborns born to mothers with infection during delivery and not in the course of gestation. We assume that this uncommon observation in our study might have been caused by the fact that during the COVID-19 pandemic, pregnant women spent more time at home and did not go to work to avoid getting infected and were more cautious and better looked after. In normal circumstances, they would have been more active and maybe less caring. They caught infection only at the end of their pregnancy, which led to other complications described in our study but not to IUGR. We plan to separately analyze the newborns born to mothers who suffered from SARS-CoV-2 infection in earlier weeks of pregnancy and not just at the time of delivery. The results in such a cohort might be completely different. It would also be interesting to analyze the newborns born to infected and non-infected mothers at the same time. However, we thought that the pandemic in such an analysis could cause bias, so we decided to select patients for a control group from the time before the pandemic.

The mean birth weight of newborns born to mothers with COVID-19 infection and of the control group was comparable. The same is described in most other studies [19,20,21].

Overall, most newborns born to mothers with a positive RT-PCR assay for SARS-CoV-2 either exhibit mild symptoms or are entirely asymptomatic. However, they may present with abnormal X-ray or chest CT scans [13]. Neonates may also present with pneumonia symptoms with cough [19]. Moreover, Wu and colleagues observed that 3 out of 30 neonates in their study were diagnosed with necrotizing enterocolitis, although they were all full-term infants [22]. Therefore, they concluded that infants born to infected mothers are at risk of developing necrotizing enterocolitis, which warrants further research and analysis. In our study, we observed some feeding intolerance in the first few days of life of infants born to mothers with SARS-Cov-2 infection. However, in all cases, this problem was only temporary and not serious.

A systematic review of newborn health performed by Duran and colleagues evaluated the outcomes of 222 neonates born to mothers with COVID-19. They reinforced that abstaining from breastfeeding or isolating newborns from their mothers was not essential [15]. Another report reported no adverse outcomes due to skin-to-skin contact, breastfeeding, or the neonate sharing rooms with the infected mother [11]. In our hospital, all infants were isolated from their mothers immediately after delivery in the first few months of the pandemic. That meant that most of them were not fed exclusively with maternal milk and the breastfeeding rate decreased. We have now changed this situation and newborns are separated from mothers only for a short time after delivery.

Our study was performed when the guidelines concerning rooming-in and breastfeeding were still unclear and changing quickly. Nowadays, the guidelines are unambiguous, and both rooming-in and breastfeeding are recommended. The evidence suggests that the risk of the newborn acquiring infection during birth hospitalization is low when precautions are taken to protect newborns from maternal infectious respiratory secretions. Mothers and newborns in good condition should be cared for using usual center practice, including rooming-in [23].

It should also be underlined that as long as an infected mother takes appropriate precautions, she should breastfeed her baby. Breastmilk is the best source of nutrition for infants, including infants whose mothers have confirmed or suspected coronavirus infection. It contains antibodies and other immunological factors that can help to protect the baby against nosocomial and household infections. It also has many benefits for future life. What is more, several recent studies have found antibodies to specific SARS-CoV-2 antigens in human milk after both maternal infection and maternal vaccination against SARS-CoV-2. Given these findings, direct breastfeeding is encouraged at this time [23,24].

## 5. Conclusions

It has been widely established that neonates born to mothers infected with SARS-CoV-2 have an overall favorable prognosis. In some cases, respiratory symptoms or distress findings may be observed. Further research and evaluation would be necessary to establish the consequences of vertical transmission of SARS-CoV-2 to neonates born to infected mothers.

Newborns born to mothers with confirmed COVID-19 should be monitored for respiratory rate, heart rate, body temperature, and gastrointestinal symptoms. Moreover, a neonatologist should always be present at the time of delivery for the possible need of resuscitation [13].

It is recommended that newborns born to mothers with COVID-19 infection should not be separated from their mothers and can be cared for in rooming-in units. Breastfeeding should also be encouraged [23,24].

## Figures and Tables

**Table 1:** The demographic data and complications of pregnancy in the study and control group.

	Covid-19 Mothers *n*= 101		Control Group *n* = 101		*p*
Gestational age (weeks) (min–max)	38.75 (34–41)		38.75 (35–41)		NS ^1^
Birth weight (g) (min–max)	3470.96 (2382–4920)		3340.74 (2160–4755)		NS ^1^
Sex					
Male	61	60.4%	63	62.4%	NS ^2^
Female	40	39.6%	38	37.6%	NS ^2^
Mode of delivery					
Cesarean section	84	83.17%	41	40.59%	***p* < 0.05 ^2^**
Natural childbirth	13	12.87%	50	49.5%	***p* < 0.05 ^2^**
Vacuum extraction	4	3.96%	10	9.9%	NS ^2^
Problems and complications during pregnancy					
Intrauterine growth restriction	1	0.99%	10	9.9%	***p* < 0.05 ^2^**
Positive group B strep test during pregnancy	14	13.86%	11	10.89%	NS ^2^
PROM	3	2.97%	4	3.96%	NS ^2^
Meconium-stained amniotic fluid	8	7.92%	12	11.88%	NS ^2^
Diabetes during pregnancy	12	11.88%	11	10.89%	NS ^2^
Hypertension during pregnancy	9	8.91%	8	7.92%	NS ^2^
Smoking during pregnancy	2	1.98%	2	1.98%	NS ^2^
Congenital anomalies	2	1.98%	3	2.97%	NS ^2^

^1^ Student’s *t*-test; ^2^ chi-square test.

**Table 2:** Comparison of the Apgar score and the need for resuscitation in the study group and control group.

	Covid-19 Mothers *n* = 101			Control Group *n* = 101			*p*
Apgar score	Median	Min.	Max.	Median	Min.	Max.	
1 st min of life	10	5	10	10	1	10	NS ^1^
5 th min of life	10	7	10	10	8	10	NS ^1^
Resuscitation in the delivery room	4	3.96%		3	2.97%		NS ^2^

^1^ U-Mann–Whitney test; ^2^ Fisher’s exact test.

**Table 3:** Different forms of respiratory distress in the study group and control group.

	Covid-19 Mothers *n* = 101		Control Group *n* = 101		*p* ^1^
Transient tachypnoea of newborns	12	11.88%	4	3.96%	***p* = 0.037**
Respiratory distress syndrome (requiring surfactant treatment)	1	0.99%	0	0%	NS
Pneumonia	3	2.97%	2	1.98%	NS
Respiratory support	16	16.0%	4	3.96%	***p* = 0.01**

^1^ chi-square test.

**Table 4:** Association between the mode of delivery and respiratory distress in the study group and control group.

		Respiratory Distress		TTN	
Covid-19 mothers	Cesarean section (*n* = 83)	15 (18.07%)	NS ^1^	12 (14.29%)	NS ^1^
	Natural childbirth (*n* = 13)	1 (7.69%)		0 (0%)	
	Vacuum extraction (*n* = 4)	0 (0%)		0 (0%)	
Control group	Cesarean section (*n* = 41)	5 (12.2%)	NS ^1^	4 (9.76%)	NS ^1^
	Natural childbirth (*n* = 50)	1 (2%)		0 (0%)	
	Vacuum extraction (*n* = 10)	0 (0%)		0 (0%)	

^1^ Fisher-Freeman-Halton test.

## Data Availability

The data presented in this study are available on request from the corresponding author.
